# Use of Apps to Promote Childhood Vaccination: Systematic Review

**DOI:** 10.2196/17371

**Published:** 2020-05-18

**Authors:** Caroline de Cock, Michelle van Velthoven, Madison Milne-Ives, Mary Mooney, Edward Meinert

**Affiliations:** 1 Digitally Enabled PrevenTative Health Research Group Department of Paediatrics University of Oxford Oxford United Kingdom; 2 School of Nursing & Midwifery Trinity College Dublin Dublin Ireland; 3 Department of Primary Care and Public Health Imperial College London London United Kingdom

**Keywords:** vaccination, vaccination coverage, mobile apps, infant, childhood vaccination, immunization, smartphone technology, mobile phone

## Abstract

**Background:**

Vaccination is a critical step in reducing child mortality; however, vaccination rates have declined in many countries in recent years. This decrease has been associated with an increase in the outbreak of vaccine-preventable diseases. The potential for leveraging mobile platforms to promote vaccination coverage has been investigated in the development of numerous mobile apps. Although many are available for public use, there is little robust evaluation of these apps.

**Objective:**

This systematic review aimed to assess the effectiveness of apps supporting childhood vaccinations in improving vaccination uptake, knowledge, and decision making as well as the usability and user perceptions of these apps.

**Methods:**

PubMed, Excerpta Medica Database (EMBASE), Web of Science, Cochrane Central Register of Controlled Trials, ClinicalTrials.gov, and Education Resources Information Center (ERIC) databases were systematically searched for studies published between 2008 and 2019 that evaluated childhood vaccination apps. Two authors screened and selected studies according to the inclusion and exclusion criteria. Data were extracted and analyzed, and the studies were assessed for risk of bias.

**Results:**

A total of 28 studies evaluating 25 apps met the inclusion criteria and were included in this analysis. Overall, 9 studies assessed vaccination uptake, of which 4 reported significant benefits (*P*<.001 or *P*=.03) of the implementation of the app. Similarly, 4 studies indicated a significant (*P*≤.054) impact on knowledge and on vaccination decision making. Patient perceptions, usability, and acceptability were generally positive. The quality of the included studies was found to be moderate to poor, with many aspects of the methodology being unclear.

**Conclusions:**

There is little evidence to support the use of childhood vaccination apps to improve vaccination uptake, knowledge, or decision making. Further research is required to understand the dichotomous effects of vaccination-related information provision and the evaluation of these apps in larger, more robust studies. The methodology of studies must be reported more comprehensively to accurately assess the effectiveness of childhood vaccination apps and the risk of bias of studies.

**International Registered Report Identifier (IRRID):**

RR2-10.2196/16929

## Introduction

### Background

In 2018, it was estimated that immunization prevented 2 to 3 million deaths each year, yet over 19 million children worldwide under the age of 1 year did not receive basic vaccines [1]. Most of these children lived in developing countries, where access to vaccines and antenatal services is somewhat limited [1]. Nevertheless, an increase in vaccine-preventable disease outbreaks has also been identified in developed countries, which is associated with declining vaccination uptake [2-4]. Immunization coverage of 9 routine childhood vaccinations declined in England by 0.2% to 1% during 2018 to 2019, compared with the previous year, and 1.3% of children born in 2015 in the United States received no vaccinations by the age of 2 years, compared with 0.9% of those born in 2011 [4,5]. A number of studies have investigated the reasons for vaccine refusal among parents and caregivers and have revealed that religious, philosophical, and personal beliefs, coupled with safety concerns and insufficient information, were the most commonly cited reasons [6]. The now-refuted evidence linking measles-mumps-rubella with autism was seen to cause a 2% decrease in the uptake of measles-mumps-rubella vaccinations [7]. Furthermore, the widespread adoption of the human papillomavirus (HPV) vaccine has been thwarted by religious and cultural barriers [8,9].

Despite the low mortality rate of vaccine-preventable diseases, various sociodemographic groups, including young children and elderly or immunocompromised individuals, are at risk of serious, sometimes fatal, complications [10]. The outbreaks of vaccine-preventable diseases can be minimized by maintaining herd immunity, which varies from 75% to 97% vaccination coverage, depending on the disease and setting in question [11]. As such, it is crucial that there is adequate provision of correct and comprehensive information, resources, and reminders to encourage parents and caregivers to obtain complete and timely vaccinations for their children. Many informational, behavioral, and environmental initiatives in various settings have been implemented to improve the uptake of childhood vaccinations [12-14]; however, the scalability and sustainability of these programs have been problematic [12].

With the increasing utilization and accessibility of mobile devices, digital technologies have shown promise in effectively disseminating information to diverse and diffuse populations and rolling out community-wide initiatives [10,15]. A World Health Organization survey on mobile health (mHealth) revealed that 83% of member states offered at least one service in 2011 [16]. mHealth has been investigated by multiple private and public organizations to support the uptake of vaccinations, including vaccination information websites and mobile apps, hereafter referred to as apps. These apps have various functions designed to support health care providers, caregivers, and, in some cases, children to access vaccine-related information, recommended immunization schedules, store vaccination records, and receive appointment reminders. There are now over 200 vaccination-related apps available on the App Store [17]. A systematic review conducted in 2015 discussing the design of vaccination reminder apps reviewed 2 studies on mobile reminder apps [18]. However, a comprehensive review of the effectiveness and usability of childhood vaccination-related apps is yet to be conducted.

### Objectives

This study aimed to systematically review the evidence on the use of apps to support childhood vaccination uptake, information storage, and record sharing as well as to investigate the usability and user perceptions of these apps.

## Methods

This systematic review was conducted following, where possible, the Cochrane collaboration [19] and the Centre for Review and Dissemination [20] methodologies for conducting systematic reviews.

### Database Search

Full methods for this review have been published in detail in a systematic review protocol [21]. This systematic review was registered with the International Prospective Register of Systematic Reviews (CRD42019156583). The Participant, Intervention, Comparison, and Outcome framework was used to develop the search strategy [22], which was performed following the Preferred Reporting Items for Systematic Reviews and Meta-Analyses Protocols [23]. The search strategy was tweaked to ensure that a defined set of known references was returned without retrieving an unmanageably large number of studies. This primarily involved the selection and amendment of wildcard terms to ensure that irrelevant terms were not included; for example, immun* retrieved papers related to immunology; hence, this was amended to immuni* to make this more specific to immunizations. No study design filter was used as both quantitative and qualitative studies were included. The search strategy was finalized and tailored to different databases in consultation with a medical librarian. PubMed, Excerpta Medica Database (EMBASE, Ovid), Web of Science, Cochrane Central Register of Controlled Trials (CENTRAL), ClinicalTrials.gov, and Education Resources Information Center (ERIC) databases were searched. The search terms were grouped into 3 themes—vaccinations, mobile apps, and children—which were subsequently searched with the following structure: vaccinations (Medical Subject Headings, MeSH OR Keywords) AND mobile applications (MeSH OR Keywords) AND children (MeSH OR Keywords). The full search strategy is shown in Multimedia Appendix 1. The search took place on October 23, 2019.

### Inclusion and Exclusion Criteria

This systematic review aimed to assess apps designed to support childhood vaccination uptake. As such, the search was limited to studies conducted during or after 2008, when the first smartphone was launched, thus reducing the number of irrelevant results. When searching ClinicalTrials.gov, the search was limited to studies first posted on or after January 1, 2008. Only studies published in English were included to ensure an accurate interpretation. Observational studies such as cross-sectional surveys, cohort studies, qualitative studies, economic studies, and intervention studies were included. Intervention studies were not required to have a specific comparator or any comparators. Studies were excluded if they were solely descriptive of the app.

To understand the latest developments in accessible technology supporting improvement in the uptake of childhood vaccinations, we restricted this review to apps hosted on mobile platforms. The app could provide any service related to the promotion of vaccination or vaccination decision making, including but not limited to information sharing and record storing or sharing, and appointment support. Studies that did not involve the use or study of an app or solely focused on other ways of delivering vaccination interventions such as text messaging, telephone calls, or web-based interventions were excluded. Owing to the specific nature of the intervention, the population was restricted to children, parents, guardians, and/or health care professionals involved in the management of children. Children were defined as individuals aged less than or equal to 18 years. Studies focusing on the vaccination of adults were excluded. The study could have been conducted in any geographical setting.

### Outcome Measures

The primary outcome of this review was the uptake of vaccination. The secondary outcomes were the knowledge and decision making of parents; costs and cost-effectiveness; use of the app; measures of usability, for example, usefulness, acceptability, and experiences of different users (parents and health care professionals); and adverse events (eg, data leak and misinformation).

### Screening and Selection of Studies

All studies retrieved from the databases were stored in Mendeley version 1.19.5 (Elsevier), a reference management software. This software automatically eliminated duplicates before screening the citations against the inclusion and exclusion criteria by 2 independent reviewers. When duplicates, or publications from the same study were identified, the more recent publication or the one with the most details was selected for inclusion in the review. Any disagreements were discussed, and if a consensus was not reached, a third reviewer was consulted.

Published results of trials that were retrieved from CENTRAL or ClinicalTrials.gov and that met the inclusion criteria were searched for and included if not already captured; trial designs or protocols were excluded. A total of 10 trials met the inclusion criteria; 8 had no published data at the date of screening, and the published results of the remaining 2 trials were already included. The titles of references of 5 relevant review studies that were retrieved with our search strategy were reviewed for inclusion; 4 additional references were identified and were included in the full-text review.

The full text of the abstracts that met the inclusion criteria was screened by one of the reviewers and validated by a second reviewer to determine the studies to be included in the final set. Overall, 10 of the screened studies eligible for inclusion were conference or meeting abstracts and did not have full texts available; hence, they were excluded.

### Data Extraction

Data were extracted by 1 reviewer, and key data points from the studies that were specified in the protocol and identified on further study of the publications were recorded in a spreadsheet. The data extraction form was based on the minimum requirements recommended by the Cochrane Handbook for Systematic Reviews [24]. The data extracted from the studies are shown in Table 1. This process was validated by a second reviewer, and disagreements were resolved by a third reviewer. There were no disagreements in the extracted data; however, there were 18 instances in which the second reviewer proposed the inclusion of additional data for greater clarity.

**Table 1:** Data extracted from the included studies.

Study information	Data extracted
General study information	Title of publication, year of publication, authors, and journal of publication
Study characteristics	Study design, country of study, analyzed sample size, key inclusion/exclusion criteria, and study arms
Intervention characteristics	App name, device on which the app could be or was utilized, compatible platforms, intended user, aim of the app, vaccines covered by the app, and vaccine-related features of the app
Evaluation	Number of users, impact on the uptake of vaccinations, impact on knowledge/learning, impact on vaccination decision making, perceived credibility, usability/user experiences, popular features, costs/cost-effectiveness, adverse events, and conclusions

### Risk of Bias

The quality assessment of the included studies was undertaken by 1 reviewer and validated by a second reviewer. Any disagreements were resolved by consensus or the opinion of a third reviewer, where required. The methods specified in the Cochrane collaboration tool for assessing the risk of bias were used. The Cochrane collaboration risk of bias tool was used to assess the quality of the randomized controlled trials [25]; the risk of bias in nonrandomized studies of interventions (ROBINS-I) tool was used to assess the nonrandomized intervention trials [26]; and the critical appraisal skills program tools for cohort, qualitative, and economic studies were used for pre-post and quantitative studies, qualitative studies, and economic studies, respectively, [27-29]. The quality of cross-sectional survey studies was assessed using the Appraisal tool for Cross-Sectional Studies (AXIS) tool [30]. The results of the Cochrane collaboration risk of bias tool and ROBINS-I evaluations were summarized using RevMan 5.3 [31] and robvis [32], respectively. The critical appraisal skills program scores were calculated using standard practice, yes=1, no=0, and cannot tell=0 for each question, following which the total score was summed for each study. AXIS scores were summarized tabularly, and the mean and SD were calculated.

### Data Analysis and Synthesis

Owing to the variability in populations, interventions, outcomes, and study designs, a meta-analysis of the studies was not possible; hence, we report a narrative overview of the findings to draw conclusions about the potential roles, value, and effectiveness of the apps to support childhood vaccinations. For the purpose of this review, the app was considered to provide significant benefit if there was a statistically significant (*P*≤.054) improvement in a given outcome as compared with a comparator or control or over time. If no significance was reported or if the difference was nonsignificant or significantly worse among groups or over time, the app was considered to have no significant evidence supporting it. The limitations and future directions for research were also summarized.

## Results

### Study Selection

**Figure 1:**
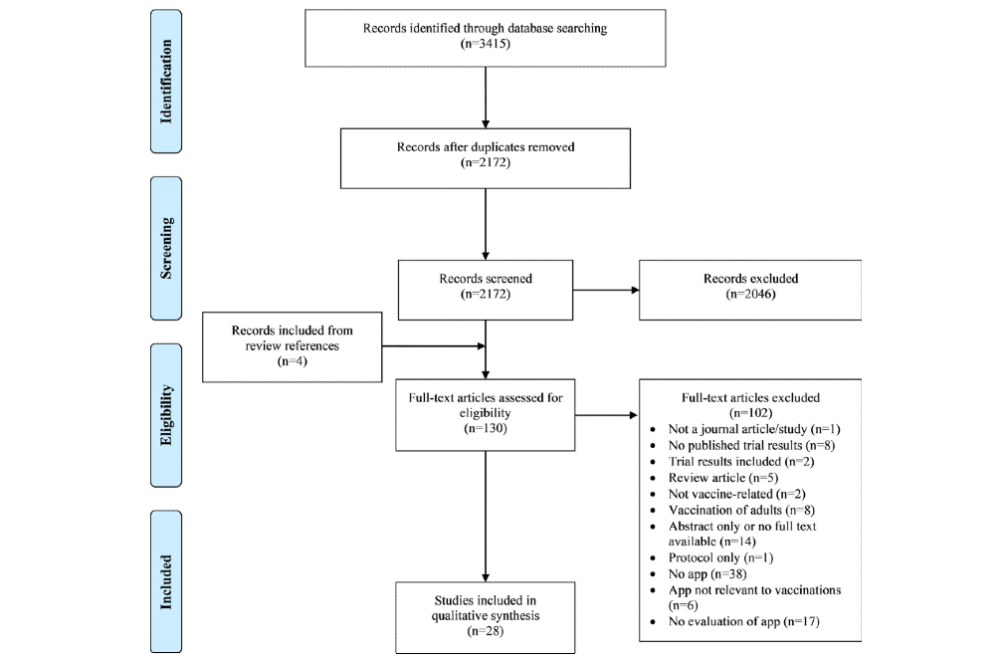
Preferred Reporting Items for Systematic Reviews and Meta-Analyses flow diagram of study selection.

Overall, 3415 studies were retrieved from the 7 databases; of these, 1243 were duplicates. Of the 2172 citations screened, 126 were selected for full-text review, and 4 additional studies were identified during the title screening of the references from 5 relevant review studies that had been retrieved in the database search. The primary reasons for exclusion at the screening stage were that the study was not vaccination-related (n=1171), did not include a mobile app (n=564), or was not health-related (n=89). Overall, 28 papers were included in the final review. The reasons for the exclusion of full-text review are detailed in Figure 1 [33].

### Study Characteristics

The study characteristics of the 28 studies included in this review can be seen in Multimedia Appendix 2. These studies were published between 2010 and 2019. Overall, 3 of the 28 studies were randomized controlled trials [34-36], and 12 had a nonrandomized trial design; 9 were pre-post (before-and-after) studies [37-45], 2 were nonrandomized controlled trials [46,47], and 1 had an interrupted time series design [48]. A further 6 studies were cross-sectional survey studies [49-54], 4 were longitudinal observational studies [55-58], 2 were qualitative studies [59], and 1 was an economic design study [60].

The 28 studies evaluated 25 unique apps; 3 papers evaluated the ImmunizeCA app [39,58,59] and 2 studies assessed MorbiQuiz [35,49]. The included studies varied in design, population, and geographical setting. The study populations generally included parents (15/28) [35-39,44,46-52,54,59], expectant mothers (5/28) [36,39,44,47,59], and/or children (4/28) [41-43,61]. Other populations included the general public (5/28) [45,53,55,57,58], households (1/28) [43], and village doctors (1/28) [34,43]. Some studies included multiple populations; hence, they were included multiple times in these statistics. The population size in the included studies ranged from 6 to 161,695 participants [43,51]. Overall, 12 of the 28 included studies were conducted in North American countries [37-40,44,45,51,53,57-60], 7 in Asia [34,36,41,42,47,52,56], 1 in the Middle East [54], 5 in Europe [35,46,49,50,61], 2 in Africa [43,48], and 1 included worldwide users [55]. Approximately, 39% (11/28) of the studies took place in deprived areas and/or developing countries [34,36,38,41-43, 47,48,52,54,56].

### App Characteristics

The characteristics of the 25 apps investigated in the included studies are shown in Table 2. The apps were primarily delivered via a smartphone or tablet, with 4 apps using multimodal delivery methods [36,37,50,56]. Over 50% (15/25) of the apps were intended for use by parents [35,37,38,40,44,46, 50-52,54-58,60], with 4 designed specifically for use by mothers [44,52,54,58]. Furthermore, 3 apps were for use by multidisciplinary populations involved in the delivery of childhood vaccinations, for example, health care providers, pharmacists, and parents [50,55,56]. Overall, 7 of the 25 apps were designed solely for the use of health care providers/health workers [34,36,41-43,47,48]. The vaccines covered by these apps varied. Almost two-thirds (15/25) of the apps covered multiple vaccines [34,36,41,42,44,46-48,50-52,54-58], 5 focused solely on HPV [37,38,40,60,61], 2 on influenza [45,53], 1 on measles-mumps-rubella [35], and 1 on measles only [43]. Furthermore, 4 studies describing 4 unique apps reported an association with the Expanded Program on Immunization (EPI) [34,41,42,47]. All 25 apps served vaccination-related functions; however, 28% (7/25) had a focus beyond vaccinations [36,42,45,50,52,54,56]. The scope of these apps remained broadly within the field of antenatal, maternal, or child health, with the exception of the Carrot Rewards app, which was intended for general health initiatives [45].

**Table 2:** Characteristics of childhood vaccination apps.

App	Intended user	Technical specifications	Compatible platforms
Conversational agent for HPV^a^ vaccination [37]	Parent	Graphical user interface tool on laptop used by an operator Bluetooth communication with iPad, the user-facing interface Text-to-speech capability Wizard of Oz agent architecture	Apple iOS^b^ and MacOS
ImmunizeCA [39,58,59]	Women of childbearing age	Generates customized vaccination schedules Vaccine information available Creates virtual immunization record Syncs with calendar for scheduling Embedded outbreak alert feature Basic security features Rotating banner in app used to display features and public health messages	iOS and Android
Tablet-based self-persuasion app [38]	Parent	Voiceover narration of task Audio recording function to facilitate self-administration	iOS
ReadyVax [38,55]	Health care providers, pharmacists, parents, and patients	Native app direct to smartphone Offline functioning Information updates automatically Browsable and searchable information Information updated through a web-based dashboard interface Alert notifications can be sent Links to multimedia	iOS
UberHealth [53]	Anyone	Request and delivery of vaccines using geolocation software	NR^c^
EPI^d^ app [34]	Doctors	Record vaccination status and upload data into the CIRS^e^ CIRS sends daily updates on children for whom vaccination is overdue Contact details of families available	NR
Carrot Rewards [45]	Anyone	In-app quiz about influenza vaccinations Geolocation-based push notification when in proximity to a pharmacist Loyalty points for completion of vaccination-related tasks	NR
MorbiQuiz [35,49]	Parent	Daily quiz targeting vaccination literacy Vaccination empowerment videos Leaderboard for quiz results	iOS and Android
Tablet-based HPV educational module [40]	Patient and parent	Educational videos on HPV Flashcard information on HPV	NR
RapidSMS [48]	Health worker	Mobile alert system for vaccination tracking	NR
CHeITA [50]	Health care providers, parents, and guardians	Stores health history of family and development statistics Vaccination tracking	Windows, iOS, and Android
Mobile technology supporting EPI coverage [41]	Health worker	Stores personal and familial information Case identification via pictures Pronunciation of the name of the child in the mother’s ethnic language	NR
Mother and Child Care Module-EPI module [42]	Health worker	Immunization status collected Connection with server module Generates appointment dates and SMS reminders	NR
Tailored interactive multimedia intervention [60]	Parent	Tailored interactive health communication about HPV via videos	NR
Baby Care app [52]	Mothers	Embedded FAQs^f^ Upload child data Trend analysis Alert messaging Baby’s periodic health report generation	NR
EpiSurveyor [43]	Health worker	Sources of information Basic demographics Consent to bring children for immunization	Android
ImTeCHO [36]	Health worker	Registration of pregnant women and children aged under 2 years Generates daily appointment schedule Videos to emphasize key health messages	Android and web
Smartphone App for Premature Infants [54]	Mothers	Electronic learning modules	NR
Call the shots [51]	Parent	Reminders for vaccination Record keeping of child’s vaccinations Hosts latest immunization schedule Extensive toolkit embedded with FAQs Links to videos and resources	Android
FightHPV [61]	Teenagers	Gamified narratives with connected text messages to convey HPV information Players able to share information with social network	iOS, Java, and Android
MomsTalkShots [44]	Mothers	Videos with obstetricians and pediatricians of different ethnicities Intervention tailored to knowledge and beliefs	NR
VaccApp [46]	Parent	Avatar requests vaccination information	Android
iCHRcloud [56]	Parent and doctor	Mobile interface, doctor module, and cloud component Child health records stored, updated, and shared across network	iOS and Android
mTika [47]	Health worker	Registration of pregnant women SMS birth notifications from mothers Automated SMS vaccination reminders to mothers and health workers EPI monitoring by supervisors	Android
iPhone app [57]	Parent	Stores child’s vaccination information Hosts recommended vaccination schedule Generates customized vaccination schedule	NR

^a^HPV: human papillomavirus.

^b^iOS: iPhone operating system.

^c^NR: not reported.

^d^EPI: Expanded Program on Immunization.

^e^CIRS: Child Immunization Register System.

^f^FAQs: frequently asked questions.

### Functionality of Childhood Vaccination Apps

The investigated apps were most commonly designed for the primary purpose of education (11/25) [35,40,43-46, 52,54,55,60,61], record keeping (8/25) [34,36,42,47,50,56-58], or reminder systems (3/25) [41,48,51], as shown in Table 3. Despite specifying distinct primary functions, the apps had multiple overlapping capabilities. On average, the apps performed 1.9 functions (range 1-4), with the most commonly occurring functions aligning with the primary functions: education (14/25), management of records (12/25), and reminders (11/25).

There was no consistent reporting of the most popular features, perceptions, or usage of individual functions. A total of 5 studies reporting on the usage of the apps noted that the most commonly used/most popular features were those that helped manage vaccination records [39], provided vaccination information [55], supported appointment management [34,59], and generated summary reports [52]. These functions aligned with the primary functions of the app being investigated. A qualitative study supplemented with Google Analytics data for the ImmunizeCA app reflected the overall data. These researchers reported that 9 of the 10 women interviewed used the vaccination tracking function, 80% used the appointment reminders/calendar, and 80% used the information on vaccines [59]. This was echoed by the Google Analytics data that were reported in the same study, wherein 47.6% of all app sessions accessed the tracking function, compared with 9.5% and 4.9%, where the appointment reminders and vaccination information were accessed, respectively [59]. One study that asked participants about the helpfulness of specific features of the app reported that all 6 respondents found the date reminder system helpful, whereas 5 respondents found the vaccine information very helpful [51].

**Table 3:** Capabilities of the apps described in the included studies.

App	Counseling	Self-persuasion	Management of records	Reminders	Vaccine-preventable disease breakout alert	Education	Frequently asked questions	Vaccine delivery	Total
Conversational agent for HPV^a^ vaccination [37]	X^b^	N/A^c^	N/A	N/A	N/A	N/A	N/A	N/A	1
ImmunizeCA [39,58,59]	N/A	N/A	X^b^	X^d^	X^d^	N/A	X^d^	N/A	4
Tablet-based self-persuasion app [38]	N/A	X^b^	N/A	N/A	N/A	X^d^	N/A	N/A	2
ReadyVax [55]	N/A	N/A	N/A	N/A	X^d^	X^b^	X^d^	N/A	3
UberHealth [53]	N/A	N/A	N/A	N/A	N/A	N/A	N/A	X^b^	1
EPI^e^ app [34]	N/A	N/A	X^b^	X^d^	N/A	X^d^	N/A	N/A	3
Carrot Rewards [45]	N/A	N/A	N/A	X^d^	N/A	X^b^	N/A	N/A	2
MorbiQuiz [35,49]	N/A	N/A	N/A	N/A	N/A	X^b^	N/A	N/A	1
Tablet-based HPV educational module [40]	N/A	N/A	N/A	N/A	N/A	X^b^	N/A	N/A	1
RapidSMS [48]	N/A	N/A	N/A	X^b^	N/A	N/A	N/A	N/A	1
CHeITA [50]	N/A	N/A	X^b^	N/A	N/A	N/A	N/A	N/A	1
Mobile technology supporting EPI coverage [41]	N/A	N/A	X^d^	X^b^	N/A	X^d^	N/A	N/A	3
Mother and child care module-EPI module [42]	N/A	N/A	X^b^	X^d^	N/A	N/A	N/A	N/A	2
Tailored interactive multimedia intervention [60]	N/A	N/A	N/A	N/A	N/A	X^b^	N/A	N/A	1
Baby Care app [52]	N/A	N/A	X^d^	X^d^	N/A	X^b^	X^d^	N/A	4
EpiSurveyor [43]	N/A	N/A	X^d^	N/A	N/A	X^b^	N/A	N/A	2
ImTeCHO [36]	N/A	N/A	X^b^	N/A	N/A	N/A	N/A	N/A	1
Smartphone App for Premature Infants [54]	N/A	N/A	N/A	N/A	N/A	X^b^	N/A	N/A	1
Call the shots [51]	N/A	N/A	X^d^	X^b^	N/A	N/A	X^d^	N/A	3
FightHPV [61]	N/A	N/A	N/A	N/A	N/A	X^b^	N/A	N/A	1
MomsTalkShots [44]	N/A	N/A	N/A	N/A	N/A	X^b^	N/A	N/A	1
VaccApp [46]	N/A	N/A	N/A	N/A	N/A	X^b^	N/A	N/A	1
iCHRcloud [56]	N/A	N/A	X^b^	X^d^	N/A	N/A	N/A	N/A	2
mTika [47]	N/A	N/A	X^b^	X^d^	N/A	N/A	N/A	N/A	2
iPhone app [57]	N/A	N/A	X^b^	X^d^	X^d^	N/A	N/A	N/A	3
Total	1	1	12	11	3	14	3	1	N/A

^a^HPV: human papillomavirus.

^b^X: indicates primary functions.

^c^N/A: not applicable.

^d^X: indicates secondary functions.

^e^EPI: Expanded Program on Immunization.

### Uptake of Vaccinations

The extracted outcomes and results are provided in Multimedia Appendix 3. Overall, 9 of the 28 studies assessed the impact of an app on vaccination uptake. Furthermore, 4 studies reported a significant improvement in vaccination coverage after versus before the implementation of the app [34,42,45,47]. These studies reported a 17% (*P*=.03) [34], 5% (*P*<.001) [45], 9.7% (*P*<.001) [42], and 17.9% (rural) and 16.4% (urban; *P*<.001 for both) [42,47] increase in the vaccination rates after versus before the implementation of the app. In addition, 2 of the 4 studies included a control group. The study of the mTika app identified a significant difference-in-difference estimate of the difference between the intervention groups’ change from baseline to end line and the control groups’ change from baseline to end line of fully vaccinated children (21.6% rural and 23.2% urban difference; *P*<.05) [47]. Conversely, the study of the EPI app did not find a significant difference between the intervention and control groups at the end line (2.5% difference; *P*=.16).

Of the remaining 5 studies, 2 reported no significant benefit [36,48] and 3 reported no significance level [41,43,53] regarding an increase in the vaccination rates between the intervention and control groups or after versus before intervention implementation.

### Vaccination Knowledge and Decision Making

A total of 10 studies reported on the impact of the vaccination apps on knowledge/learning, as shown in Multimedia Appendix 3. Furthermore, 4 studies reported significant improvements in the knowledge or learning compared with a control group or after versus before the intervention (*P*≤.05) [35,40,46,61], 2 reported no significant improvement [39,41], and 4 did not indicate a significance level [43,47,49,54].

The implications of the vaccination apps on decision making and evaluation of the risk-benefit of vaccinations were investigated in 8 studies, as shown in Multimedia Appendix 3. Furthermore, 7 of the 8 studies reporting this outcome indicated a positive impact of the apps on vaccination beliefs and intent to vaccinate [35,38-41,44,49]. The remaining study was unable to report improvements because of no vaccine hesitancy in participants at baseline [37]. Half of the studies (4/8) indicated significant improvements (*P*≤.05) in the intent to vaccinate children, positive attitudes toward vaccination, and/or confidence in their vaccination decision after interaction with the app versus before or compared with a control group [35,38,40,41]. The remaining 4 studies did not report a significance level [37,39,44,49]. Fadda et al [35] investigated the knowledge and empowerment functions separately and found that exposure to the knowledge intervention significantly improved the intent to vaccinate (*P*=.03) and confidence in the participants’ vaccination decision (*P*=.006) versus control, but empowerment and combined interventions did not show stronger intent to vaccinate. Furthermore, 3 studies indicated that as well as having the potential to promote vaccination, the apps also had the potential to discourage users from vaccinating their children [39,44,49].

### Costs/Cost-Effectiveness

Only 1 study reported on the costs or cost-effectiveness of a childhood vaccination app. The cost of developing a computerized, tailored, interactive multimedia intervention was found to be approximately double the cost of a print-based Photonovella intervention for HPV vaccine education (US $135,978 vs US $66,468, respectively). This difference was retained in amortized annual costs over a 7-year period (US $21,825 vs US $10,669 per year for the tailored, interactive multimedia intervention and Photonovella, respectively) [60].

### Usability and Acceptability

Overall, 9 studies reported on the usability/ease of use (n=5), acceptability (n=1), or both (n=3) aspects of the vaccination apps. Furthermore, 8 of these studies reported high ease of use (average score for ease of use/usability >70%, or >70% of the participants rated the app easy to use). A total of 2 studies also reported high acceptance of the app (average score for acceptance >70%, or >70% of participants reporting acceptance).

### Participant Perceptions

A total of 11 studies reported on participants’ perceptions of childhood vaccination apps. Furthermore, 9 studies reported on the perceptions of parents [38-40,44,46,49,51,52,54], 1 reported on teenagers’ experiences [61], and 1 reported on the perceptions of mothers and vaccination service providers [47]. All 10 studies reporting quantitative results indicated positive user experiences, with participants considering the app to be helpful and/or trustworthy or reporting that they were satisfied, confident, and/or likely to recommend the app (average score of >70%, or >70% of the participants agreeing with relevant statements) [38-40,44,46,49,51,52,54,61]. The study reporting on the qualitative experiences of service providers and mothers with the mTika app revealed that the app was perceived as helpful, easily understood by mothers, user-friendly, time-efficient, and helpful in reducing the workload of vaccination service providers [47].

### Risk of Bias Assessment

Owing to the heterogeneity of the study types, a variety of quality assessment tools were employed to assess the risk of bias for the 28 included studies. The summary tables and figures are provided in Multimedia Appendix 4. Overall, the quality of the studies assessed in this review ranged from moderate to poor. The studies assessed using the critical appraisal skills program cohort, qualitative, and economic checklists met on average 6.4 out of 12 (range 4-9), 6.5 out of 10 (range 6-7), and 9 out of 12 (range 9) criteria, respectively. Cross-sectional studies assessed using the AXIS tool had a mean score of 9.3 (SD 2.2) out of 20. Over 50% (15/28) of the studies were deemed to have inappropriate recruitment strategies [34,36,37,41,42,45,46,48,49,52,54,56,58,61], primarily owing to the lack of sufficient information. Risk of bias in exposure (performance bias) was identified in 13 out of 17 studies in which this was assessed [34-37,41,43-45,48,55-58]. Outcome bias (detection bias) was suspected in 5 out of 19 studies in which it was assessed [34,36,41,42,48]. The implications/value of the research and fit of the results in context was lacking in 11 out of 17 [37,39,42,44,48,55-60] and 7 out of 15 [37,40,43-45,56,60] studies assessed for these criteria, respectively.

The critical appraisal skills program cohort checklist assessed confounding, completeness, and the duration of follow-up. The identification and mitigation of confounders was not found to be sufficient in any of the 14 studies assessed using this checklist [37-42,44,45,48,55-58]. A total of 13 studies gave no information on confounders, and the remaining study noted that despite the identification of potential confounders, they were unable to perform fixed or random-effects modeling to assess the impact of these factors [38]. Data on dropout rate were not recorded systematically (2/14) [45,58]; this was primarily because of a lack of substantiation of follow-up duration, information on the timing of follow-up, and/or no indication of whether discontinuations were significant.

Six cross-sectional studies assessed using the AXIS tool were found to lack justification of sample size, definition of target population (1/6), categorization of (1/6) [50,51] and information on (0/6) nonresponders, validation of outcome measures (2/6) [52,54], repeatability (2/6) [49,51], and internal consistency of results (2/6) [51]. Most of these shortcomings were because of a lack of information. Moreover, 1 of the 2 nonrandomized controlled studies was deemed to have an overall serious risk of bias because of the serious risk of selection bias in determining the intervention groups [46].

## Discussion

### Principal Findings

In this systematic review, 28 studies evaluating 25 childhood vaccination apps were examined. Overall, there is little evidence to suggest that childhood vaccination apps are effective in improving vaccination coverage, with only 4 of the 9 studies assessing this outcome indicating significant benefit (*P*≤.05) after versus before the app was introduced. This contrasts with a systematic review and meta-analysis conducted by Harvey et al [14], which revealed a significant benefit of reminder (*P*<.001), recall (*P*=.02), reminder and recall (*P*<.006), and educational initiatives on childhood vaccination rate (*P*=.02); however, these prompts were not delivered via the mobile app. Our findings also contrast with systematic reviews that have found mobile apps to be effective in eliciting health-related behavior change [62,63].

Similarly, 4 out of 10 studies assessing the impact on vaccination knowledge and 4 out of 8 studies assessing vaccination decision making reported significant benefit of the app (*P*≤.05). Furthermore, 3 studies substantiated the dichotomous effect of information provision, thus illustrating the potential for apps to dissuade individuals from vaccination. This is in keeping with evidence from other studies [64,65]. Parental decision making regarding childhood vaccination is understood to be a specific scenario for health-related decision making where parents have been stipulated to put major weight on the subjective perception of the outcome [66]. It is, therefore, important to understand the likely interpretation of the information provided and how this may affect the parental risk-benefit analysis of vaccination.

The primary functionality of the apps described in the included studies varied; however, most had multiple functions, with the most common features being education, reminders, and record keeping. These were primarily for the use of parents or health care providers; only the iCHRcloud app facilitated the sharing of vaccination record information between parents and physicians [56]. No apps had been designed for the use of school staff, and no apps had the functionality to share information between schools and parents. In the United Kingdom, many childhood and adolescent vaccinations are delivered at schools for convenience and to enhance delivery [67,68]. This could be an avenue for further investigation.

There is insufficient evidence to draw any conclusions regarding the relationship between the function of the app and the efficacy in improving vaccination rates, knowledge, or positive decision making. The 25 investigated apps had diverse functionalities but were primarily designed for providing vaccine information and/or record keeping. Studies reporting on user statistics revealed that the most popular functions were record keeping, reminders, and information access. Overall, usability, acceptability, and user perceptions of the apps were positive.

### Quality of the Evidence

The quality assessment of the included studies revealed that many were of poor to moderate quality, indicating an overall high risk of bias, which risks impairing the validity of the conclusions regarding the effectiveness of childhood vaccination apps. One study was determined to have a serious risk of bias. Most negative indicators were because of a lack of information about the criteria assessed. The risk of bias and inadequate robustness may be because of the nature of many of the included studies being pilot, early usability, and preliminary scoping studies. To draw valid and accurate conclusions on the quality of studies, study methods should be comprehensively reported. The infancy of these types of apps also had an impact on the assessment of the implications/value and the fit of the results in context, as many studies indicated that they were the first of their kind in their setting.

### Strengths and Weaknesses of the Study

The strengths of this study lie in the comprehensive analysis of the available literature discussing apps for childhood vaccination. We investigated ClinicalTrials.gov and ERIC databases, which include gray literature, and we included letters and full-text conference proceedings [50,53]. The inclusion of gray literature minimizes publication bias and ensures that a comprehensive view of the latest literature is reported [69]. We also included all study types, thereby ensuring that apps at every stage of development were reported; a study limited to randomized controlled trials may omit studies of apps in their infancy. One limitation of this review is because of the heterogeneity of the studies and their reported outcomes; therefore, it was not possible to conduct a meta-analysis. A meta-analysis would enable the quantification of the heterogeneity of studies and allow us to quantify the effectiveness of childhood vaccination apps.

### Implications of the Study

Immunization is a simple and effective mechanism for reducing childhood mortality. Despite the insignificant findings of this review about the effect of apps on the uptake of vaccinations, the positive user perceptions, usability, and acceptability reported present a compelling opportunity to build on the successes of current apps and learn from their shortcomings. Individual studies included in this review reported the potential benefit of these apps on an individual, community, and nationwide level, highlighting the breadth of engagement that can be harnessed with the use of mobile apps [42,45,47]. It is evident that more needs to be done to ensure that a positive change in vaccination knowledge and decision making is paralleled with the provision of sufficient, accessible resources to allow vaccinations to be easily obtained. There are important aspects to consider with regard to the needs of users and how apps will be implemented in health care service delivery.

### Unanswered Questions and Future Research

Mobile apps will likely play a role in the storage and sharing of vaccination records, generation of reminders, and/or dissemination of vaccination education. Despite several publicly available apps and others in development, a lack of robust evidence remains regarding the effectiveness of vaccination apps in improving vaccination coverage. This systematic review, despite not reporting significant efficacy, indicates that many of the apps convey some degree of benefit with regard to improving vaccination uptake, knowledge, and/or decision making and are widely accepted by their users. For future research, it will be important to understand the priorities of different user groups in terms of app functionalities and the dichotomous effects of vaccination information. Many of the studies included in this review are early-stage investigations and were found to have a relatively high risk of bias. Further investigation of these apps in larger, more robust, controlled trials will allow greater granularity of evaluation and understanding of the role and implications of these apps for wider communities and various subpopulations. In addition, many of the included studies originated from developing countries; however, preventable childhood illnesses are increasing globally. Outcomes from similar studies in developed countries may present a different picture.

### Conclusions

The objective of this systematic review was to evaluate the effectiveness of childhood vaccinations in improving vaccination uptake, knowledge, and decision making as well as to investigate the usability and patient perceptions of these interventions. Overall, 28 studies describing 25 apps were investigated. Although the apps were generally positively received and had high usability and acceptability scores, there was little evidence to suggest that they were effective in significantly improving uptake, knowledge, or decision making; however, most apps were seen to provide some benefit. This indicates that there is demand and engagement with apps supporting childhood vaccinations; however, further investigation is required.

The studies investigating these apps were considered to be of poor to moderate quality, likely because of the early phase nature of many of the apps and their respective studies. Only 5 studies were randomized. An additional concern raised by 3 studies was the potential for these apps to discourage vaccination among those who were initially undecided about infant vaccination and among those who had previously intended to vaccinate their children. Future research is warranted into the dichotomous effects of the provision of vaccination information, the outcomes of larger robust studies of these apps, and the needs and priorities of various user populations.

## Appendix

Multimedia Appendix 1 Search strategy.

Multimedia Appendix 2 Study characteristics.

Multimedia Appendix 3 Study outcomes and extracted effectiveness results.

Multimedia Appendix 4 Risk of bias assessment.

## Abbreviations

AXIS: Appraisal tool for Cross-Sectional Studies
CENTRAL: Cochrane Central Register of Controlled Trials
EPI: Expanded Program on Immunization
ERIC: Education Resources Information Center
HPV: human papillomavirus
MeSH: Medical Subject Headings
mHealth: mobile health
ROBINS-I: risk of bias in nonrandomized studies of interventions
