# Supplementary material for: Use of Apps to Promote Childhood Vaccination: Systematic Review
Source: JMIR Mhealth Uhealth. 2020 May 18;8(5):e17371. doi: 10.2196/17371 (PMC7265109; doi:10.2196/17371)
Supplement: Multimedia Appendix 1 [file mhealth_v8i5e17371_app1.docx]

**Appendices**

**Appendix A: Search strategy**

| **Database** | **Search terms** |
| --- | --- |
| PubMed | (Vaccines/ OR Immunization/ OR vaccin* OR Immuni* OR inoculat* OR boost*) AND (Telemedicine/ or Smartphone/ or Cell phone/ or Reminder systems/ or Mobile applications/ or smartphone* OR "mobile phone" OR "reminder system" OR "eHealth" OR "text reminder" OR "mHealth" OR "mobile health" OR mobile app* OR app OR apps OR smartphone app* OR tablet OR "mobile education" OR "electronic reminder") AND (Child/ OR baby/ OR child OR children OR childhood OR pediat* OR paediat* OR infant* OR school* OR newborn OR toddler OR baby OR juvenile OR youth OR preschool* OR adolescen* OR family OR families OR parent* OR carer OR caregiver OR kindergarten OR nursery) |
| Medline (OVID) | (Vaccines/ or Immunization/ or vaccin*.mp. or Immuni*.mp. or inoculat*.mp. or boost*.mp.) AND (Telemedicine/ or Smartphone/ or Cell phone/ or Reminder systems/ or Mobile applications/ or smartphone*.mp. or "mobile phone".mp. or "reminder system".mp. or "eHealth".mp. or "text reminder".mp. or "mHealth".mp. or "mobile health".mp. or mobile app*.mp. or app.mp. or apps.mp. or smartphone app*.mp. or tablet.mp. or "mobile education".mp. or "electronic reminder".mp.) AND (Child/ or baby/ or child.mp. Or children.mp. Or childhood.mp. or pediat*.mp. or paediat*.mp. or infant*.mp. or school*.mp. or newborn.mp. or toddler.mp. or baby.mp. or juvenile.mp. or youth.mp. or preschool*.mp. or adolescen*.mp. or family.mp. or families.mp. or parent*.mp. or carer.mp. or caregiver.mp. or kindergarten.mp. or nursery.mp.) |
| Embase (OVID) | (vaccine/ or immunization/ or "vaccin*".mp. or "Immuni*".mp. or inoculat*.mp. or boost*.mp.) AND (Telemedicine/ or smartphone/ or mobile phone/ or reminder system/ or mobile application/ or smartphone*.mp. or "mobile phone".mp. or "reminder system".mp. or "eHealth".mp. or "text reminder".mp. or "mHealth".mp. or "mobile health".mp. or "mobile app*".mp. or app.mp. or apps.mp. or "smartphone app*".mp. or tablet.mp. or "mobile education".mp. or "electronic reminder".mp.) AND (Child/ or baby/ or child*.mp. or pediat*.mp. or paediat*.mp. or infant*.mp. or school*.mp. or newborn.mp. or toddler.mp. or baby.mp. or juvenile.mp. or youth.mp. or preschool*.mp. or adolescen*.mp. or family.mp. or families.mp. or parent*.mp. or carer.mp. or caregiver.mp. or kindergarten.mp. or nursery.mp.) |
| Web of Science | ALL=(vaccin* OR Immuni* OR inoculat* OR boost*) AND ALL=(telemedicine or Smartphone or “Cell phone” or “Reminder systems” or “Mobile applications” or "smartphone*" OR "mobile phone" OR "reminder system" OR "eHealth" OR "text reminder" OR "mHealth" OR "mobile health" OR mobile app* OR app OR apps OR smartphone app* OR tablet OR "mobile education" OR "electronic reminder") AND ALL=(baby OR child OR children OR childhood OR pediat* OR paediat* OR infant* OR school* OR newborn OR toddler OR baby OR juvenile OR youth OR preschool* OR adolescen* OR family OR families OR parent* OR carer OR caregiver OR kindergarten OR nursery) |
| Cochrane  CENTRAL | ([mh vaccines] OR [mh immunity] OR vaccin* OR immuni* OR inoculat* OR boost*) AND ([mh telemedicine] OR [mh smartphone] OR [mh "cell phone"] OR [mh "reminder systems"] OR [mh "mobile applications"] OR smartphone* OR mobile phone OR reminder system OR eHealth OR text reminder OR mHealth OR mobile health OR mobile app* OR app OR apps OR smartphone app* OR tablet OR mobile education OR electronic reminder) AND ([mh child] OR [mh baby] OR child OR children OR childhood OR pediat* OR paediatr* OR infant* OR school* OR newborn OR toddler OR baby OR juvenile OR youth OR preschool* OR adolescen* OR family OR families OR parent* OR carer OR caregiver OR kindergarten OR nursery) |
| ClinicalTrials.gov | (vaccine OR Immunise OR inoculate OR booster) AND (telemedicine or phone or reminder or app or mHealth OR app OR tablet OR mobile education) AND (baby OR child OR paediatric OR adolescent OR family OR parent OR carer OR infant) |
| ERIC | (Vaccines OR Immunization OR vaccination OR Immunisation OR inoculate OR booster) AND (Telemedicine or Smartphone or Cell phone or Reminder systems or Mobile applications or smartphone OR "mobile phone" OR "reminder system" OR "eHealth" OR "text reminder" OR "mHealth" OR "mobile health" OR mobile app OR app OR apps OR smartphone app OR tablet OR "mobile education" OR "electronic reminder") AND (child OR children OR childhood OR pediatric OR paediatric OR infant OR school OR newborn OR toddler OR baby OR juvenile OR youth OR preschool OR adolescent OR family OR families OR parent OR carer OR caregiver OR kindergarten OR nursery) |
