# Supplementary material for: Use of Apps to Promote Childhood Vaccination: Systematic Review
Source: JMIR Mhealth Uhealth. 2020 May 18;8(5):e17371. doi: 10.2196/17371 (PMC7265109; doi:10.2196/17371)
Supplement: Multimedia Appendix 2 [file mhealth_v8i5e17371_app2.docx]

**Appendix B: Study characteristics**

| **Authors (year)** | **Study design** | **Country  of study** | **Study population (N)** | **N (ITT)** | **App** |
| --- | --- | --- | --- | --- | --- |
| Amith et al (2019) [[37]](https://paperpile.com/c/Y5SGkE/EKPJc) | Pre-post | USA | Parents with ≥1 child aged <18 years | (18; 16 participated) | Conversational agent  for HPV vaccination |
| Atkinson et al (2016) [[39]](https://paperpile.com/c/Y5SGkE/BgvoB) | Pre-post | Canada | Mothers in their third trimester of pregnancy or who had given birth <3 months previously | 50 | ImmunizeCA |
| Baldwin et al (2017) [[38]](https://paperpile.com/c/Y5SGkE/77zdz) | Pre-post | USA | Parents of ≥1 child aged 11-17 years | 45 | Tablet-based self-persuasion app |
| Bednarczyk et al (2014) [[55]](https://paperpile.com/c/Y5SGkE/TmraJ) | Longitudinal | Worldwide | Anyone | 5,142 | ReadyVax |
| Brownstein et al (2015) [[53]](https://paperpile.com/c/Y5SGkE/Worce) | Cross-sectional | USA | Anyone in Boston, New York City, Washington DC or Chicago | 2,378; 486 responded to survey | UberHealth |
| Burgess et al (2016) [[59]](https://paperpile.com/c/Y5SGkE/YoGAB) | Qualitative | Canada | Mothers in their third trimester of pregnancy or who had given birth <3 months previously | 10 | ImmunizeCA |
| Chen et al (2016) [[34]](https://paperpile.com/c/Y5SGkE/FZT4X) | RCT | China | Village doctors in charge of childhood vaccination | 32; 16 matched pairs | EPI app |
| Dale et al (2019) [[45]](https://paperpile.com/c/Y5SGkE/57EGy) | Pre-post | Canada | Users of the Carrot Rewards app (aged ≥13 years) | 80,229 | Carrot Rewards |
| Fadda et al (2017) [[35]](https://paperpile.com/c/Y5SGkE/Zo1jj) | RCT | Italy | Parents of ≥1 child born after 1/9/2015 | 184; Knowledge intervention: 48, Empowerment intervention: 45, Combined intervention: 47, Control: 44 | MorbiQuiz |
| Fadda et al (2018) [[49]](https://paperpile.com/c/Y5SGkE/nRN7e) | Cross-sectional | Italy | Parents of ≥1 child aged <15 months | 140; Knowledge intervention: 48, Empowerment intervention: 45, Combined intervention: 47, Control: 44 | MorbiQuiz |
| Gockley et al (2019) [[40]](https://paperpile.com/c/Y5SGkE/RYhCc) | Pre-post | USA | Patients attending colposcopy clinic | 119 | Tablet-based HPV educational module |
| Hategeka et al (2019) [[48]](https://paperpile.com/c/Y5SGkE/cm7ju) | Interrupted time series | Rwanda | Women aged 15-49 who had given birth within 5 years | NR | RapidSMS |
| Heavin et al (2014) [[50]](https://paperpile.com/c/Y5SGkE/IeukT) | Cross-sectional | Ireland | Parents of ≥1 child aged <1 year | 55 | CHeITA |
| Kaewkungwal et al (2015) [[41]](https://paperpile.com/c/Y5SGkE/QUlqu) | Pre-post | Thailand- Myanmar- Laos | Highland children aged <6 years | 3,649 | Mobile technology supporting EPI coverage |
| Kaewkungwal et al (2010) [[42]](https://paperpile.com/c/Y5SGkE/qVx8w) | Pre-post | Thailand | Children aged <6 years | 544 | MCCM-EPI module |
| Karanth et al (2017) [[60]](https://paperpile.com/c/Y5SGkE/6v4YR) | Economic | USA | N/A | N/A | Tailored interactive multimedia intervention |
| Kuo et al (2012) [[52]](https://paperpile.com/c/Y5SGkE/WmUy4) | Cross-sectional | China | Post-partum patients with full term babies | 64 | Baby Care app |
| Mbabazi et al (2015) [[43]](https://paperpile.com/c/Y5SGkE/D9Usj) | Pre-post | Kenya | Households with ≥1 child aged 9-59 months | 164,643 households; 161,695 children | Episurveyor |
| Modi et al (2019) [[36]](https://paperpile.com/c/Y5SGkE/p9kSf) | cluster-RCT | India | All pregnant women, neonates and infants | Mothers of 1-4 month old infants ITT: 1,571 intervention, 1,452 control; mothers 6-9 months old infants: 1,757 intervention, 1,713 control | ImTeCHO |
| Nourani et al (2019) [[54]](https://paperpile.com/c/Y5SGkE/zR3tA) | Cross-sectional | Iran | Mothers of premature infants | 20 | Smart Phone App for Premature Infants |
| Peck et al (2014) [[51]](https://paperpile.com/c/Y5SGkE/8X9zj) | Cross-sectional | USA | Parents of ≥1 child aged ≤18 years (6) | 6 | Call the shots |
| Ruiz-Lopez et al (2014) [[61]](https://paperpile.com/c/Y5SGkE/aHYeK) | Qualitative | Norway | Focus group 1: Individual aged 40-50 years; Focus group 2: Children aged 16-18 years | 26 | FightHPV |
| Salmon et al (2019) [[44]](https://paperpile.com/c/Y5SGkE/HD5RW) | Pre-post | USA | Women 8-26 weeks pregnant | 1,103 | MomsTalkShots |
| Seeber et al (2017) [[46]](https://paperpile.com/c/Y5SGkE/1RPHX) | Non-RCT | Germany | Parents of ≥1 child aged 0-18 years | 456; 178 intervention, 278 control | VaccApp |
| Singh et al (2018) [[56]](https://paperpile.com/c/Y5SGkE/P8bJO) | Longitudinal | India | Parents | 16,490 | iCHRcloud |
| Uddin et al (2017) [[47]](https://paperpile.com/c/Y5SGkE/hIa7H) | Non-RCT | Bangladesh | Pregnant women, mothers with children aged 0-11 months and immunisation service providers | Control 1^*^: 520/520, Intervention 1^*^: 520/520  Control 2^*^: 520/522 Intervention 2^*^: 518/520 | mTika |
| Wilson et al (2014) [[57]](https://paperpile.com/c/Y5SGkE/yfZ2t) | Longitudinal | Canada | Any Canadian | 4,867 | iPhone app |
| Wilson et al (2015) [[58]](https://paperpile.com/c/Y5SGkE/85O60) | Longitudinal | Canada | Any Canadian | 67,203 | ImmunizeCA |

^*^N numbers reported baseline/endline. EPI, expanded programme on immunization; HPV, human papillomavirus; ITT, intention-to-treat; N/A, not applicable, NR, not reported; RCT, randomised controlled trial.
