# Supplementary material for: Use of Apps to Promote Childhood Vaccination: Systematic Review
Source: JMIR Mhealth Uhealth. 2020 May 18;8(5):e17371. doi: 10.2196/17371 (PMC7265109; doi:10.2196/17371)
Supplement: Multimedia Appendix 3 [file mhealth_v8i5e17371_app3.docx]

**Appendix C: Study outcomes and extracted effectiveness results**

| **Authors (year)** | **N (ITT)** | **Outcomes** | **Uptake of vaccinations** | **Knowledge/ learning** | **Vaccine decision-making** |
| --- | --- | --- | --- | --- | --- |
| Amith et al (2019) [[37]](https://paperpile.com/c/Y5SGkE/EKPJc) | (18; 16 participated) | - Usability - Vaccine hesitancy - Features and requirements for feasibility | NR | NR | Change in vaccine hesitancy could not be measured as no hesitance was reported at baseline |
| Atkinson et al (2016) [[39]](https://paperpile.com/c/Y5SGkE/BgvoB) | 50 | - Attitudes, beliefs and behaviours regarding paediatric vaccination | NR | NR | 32% perceived the app made them more likely to vaccinate on time. 14% were more in agreement that vaccinating on time was important; 22% became less convinced |
| Baldwin et al (2017) [[38]](https://paperpile.com/c/Y5SGkE/77zdz) | 45 (33 were against or had not decided on vaccination at baseline) | - Feasibility - Vaccination decision-making | NR | NR | 81.8% (27/33) decided in favour of vaccination, 15.1% (5/33) remained undecided and 3% (1/33) decided against; p<0.001 decided vs undecided or against |
| Bednarczyk et al (2014) [[55]](https://paperpile.com/c/Y5SGkE/TmraJ) | 5,142 | - Utilization | NR | NR | NR |
| Brownstein et al (2015) [[53]](https://paperpile.com/c/Y5SGkE/Worce) | 2,378; 486 responded to survey | - Vaccination uptake - Preferences regarding delivery | 2057 persons were vaccinated; 19.8% of respondents were definitely not likely to receive vaccines from traditional providers | NR | NR |
| Burgess et al (2016) [[59]](https://paperpile.com/c/Y5SGkE/YoGAB) | 10 | - Utilization - Usability | NR | NR | NR |
| Chen et al (2016) [[34]](https://paperpile.com/c/Y5SGkE/FZT4X) | 32; 16 matched pairs | - Vaccination coverage - User perceptions | Full vaccination increased by 17% (p=0.028) and 10% (p=0.014) in the intervention group and control group, respectively; p=0.164 between intervention and control at endline | NR | NR |
| Dale et al (2019) [[45]](https://paperpile.com/c/Y5SGkE/57EGy) | 80,229 | - Uptake of flu campaign - Uptake of flu vaccine | ~5% more users received the influenza vaccine during the 2016 influenza season than in 2015 (p<0.001) | NR | NR |
| Fadda et al (2017) [[35]](https://paperpile.com/c/Y5SGkE/Zo1jj) | 184; Knowledge: intervention: 48, Empowerment intervention: 45, Combined intervention: 47, Control: 44 | - Vaccination knowledge - Empowerment - Risk perception - Preferred decisional role - Vaccination intention, attitude, confidence and recommendation | NR | Gain in vaccination knowledge in all experimental groups vs control (p<0.000) | Knowledge group more likely to vaccinate than control (p=0.03); Combined and empowerment groups did not show significant increase in likelihood to vaccinate vs control |
| Fadda et al (2018) [[49]](https://paperpile.com/c/Y5SGkE/nRN7e) | 140;  Knowledge: intervention: 48, Empowerment intervention: 45, Combined intervention: 47 | - Usability - User experience | NR | Mean score for increased knowledge of vaccine: 3.89-4.70/5 (77.8%-94.0%) | 86.5% reported the app could make parents opt for vaccination; 12.1% that it would make no difference and 1.4% that it discouraged vaccination |
| Gockley et al (2019) [[40]](https://paperpile.com/c/Y5SGkE/RYhCc) | 119 | - Vaccination knowledge | NR | Significant improvement in knowledge of the connection between HPV and cervical cancer (p=0.05), head and neck cancer (p<0.001), abnormal Pap tests (p=0.01), breast cancer [incorrect] (p=0.05) and genital warts (p=0.04) | Significant change in the likelihood of vaccinating their children (p=0.05); 95.5% vs 99.1% pre vs post use of app |
| Hategeka et al (2019) [[48]](https://paperpile.com/c/Y5SGkE/cm7ju) | NR | - Uptake of antenatal care, health facility delivery and vaccination coverage | 0.58% change (BCG) and -0.75% change (Polio) post vs pre RapidSMS intervention (p=0.2 and p=0.7, respectively) | NR | NR |
| Heavin et al (2014) [[50]](https://paperpile.com/c/Y5SGkE/IeukT) | 55 | - User perceptions | NR | NR | NR |
| Kaewkungwal et al (2015) [[41]](https://paperpile.com/c/Y5SGkE/QUlqu) | 3,649 | - Vaccination coverage - Knowledge, attitudes and practice | Vaccine coverage for polio and DTP increased from 91.7% to 94.4%. Fluctuations in monthly coverage suggested a slightly higher trend after vs before app use | Knowledge scores increased at 6 and 12 months but not significantly (p=0.134) | Positive attitude to vaccination increased over the implementation period (p=0.004) |
| Kaewkungwal et al (2010) [[42]](https://paperpile.com/c/Y5SGkE/qVx8w) | 544 | - Uptake of antenatal services and EPI visits | 44.22% of children received scheduled vaccines on-time after vs 34.49% before implementation of the MCCM (p<0.001) | NR | NR |
| Karanth et al (2017) [[60]](https://paperpile.com/c/Y5SGkE/6v4YR) | N/A | - Costs of development | NR | NR | NR |
| Kuo et al (2012) [[52]](https://paperpile.com/c/Y5SGkE/WmUy4) | 64 | - User perceptions | NR | NR | NR |
| Mbabazi et al (2015) [[43]](https://paperpile.com/c/Y5SGkE/D9Usj) | 164,643 households; 161,695 children | - Vaccination uptake | Pre-campaign visits showed 42 041 children were not going to be brought to vaccination vs 170 post-campaign | Lack of knowledge as a reason for not bringing children for vaccination did not significantly reduce post- vs pre-campaign | NR |
| Modi et al (2019) [[36]](https://paperpile.com/c/Y5SGkE/p9kSf) | Mothers (child 1-4 months): intervention: 1,571; control: 1,452. Mothers (child 6-9 months): intervention: 1,757; control: 1,713 | - Uptake of antenatal services | No difference in children that received all three doses of pentavalent vaccine between intervention and control clusters at endline (73.0% vs 73.6%) p=0.589 | NR | NR |
| Nourani et al (2019) [[54]](https://paperpile.com/c/Y5SGkE/zR3tA) | 20 | - Usability | NR | Mean score for learning 8.13/10 (81.3%) | NR |
| Peck et al (2014) [[51]](https://paperpile.com/c/Y5SGkE/8X9zj) | 6 | - Feasibility - User perceptions | NR | NR | NR |
| Ruiz-Lopez et al (2014) [[61]](https://paperpile.com/c/Y5SGkE/aHYeK) | 26 (4 focus group 1; 22 focus group 2) | - User perceptions - Knowledge | NR | All 22 from focus group 2 showed improvement in knowledge of epithelial cells (p=0.001), HPV (p=0.001), the HPV vaccine (p=0.006), HPV transmission (p=0.006) and screening (p=0.01) compared to prior to engaging with the app | NR |
| Salmon et al (2019) [[44]](https://paperpile.com/c/Y5SGkE/HD5RW) | 1,103 | - User perceptions - Knowledge | NR | 95% reported having enough information to decide about vaccines for infants after watching the videos | 72% of those without sufficient information to decide on infant vaccination at baseline reported sufficient information after watching the videos. 2% of those with enough information pre-videos did not have enough after the videos |
| Seeber et al (2017) [[46]](https://paperpile.com/c/Y5SGkE/1RPHX) | 456; 178 intervention, 278 control | - Knowledge (recall) | NR | 24.7% of parents were able to answer all three sample questions correctly before vs 63.5% after VaccApp utilization (p <0.0001) | NR |
| Singh et al (2018) [[56]](https://paperpile.com/c/Y5SGkE/P8bJO) | 16,490 | - Utilization | NR | NR | NR |
| Uddin et al (2017) [[47]](https://paperpile.com/c/Y5SGkE/hIa7H) | Control 1^*^: 520/520, Intervention 1^*^: 520/520≤  Control 2^*^: 520/522 Intervention 2^*^: 518/520 | - Vaccination uptake | Full vaccination increased from 58.9% to 76.8% and 40.7% to 57.1% in the rural and urban intervention areas, respectively (both p<0.001) and decreased in the rural and urban control areas from 65.9% to 55.2% and 44.5% to 59.9%, respectively (both p<0.001); rural DID p<0.001 and urban DID p<0.05 | Mean score for ease of learning 4.14/5 (82.8%) | NR |
| Wilson et al (2014) [[57]](https://paperpile.com/c/Y5SGkE/yfZ2t) | 4,867 | - Utilization | NR | NR | NR |
| Wilson et al (2015) [[58]](https://paperpile.com/c/Y5SGkE/85O60) | 67,203 | - Utilization | NR | NR | NR |

BCG, Bacillus Calmette-Guerin; DID, difference-in-difference; DPT, diphtheria, pertussis and tetanus; HPV, human papillomavirus; ITT, intention-to-treat; MCCM, Mother and Child Care Module; NR, not reported; WHO-ICV, World Health Organization International Certificate of Vaccination.
