# Supplementary material for: Use of Apps to Promote Childhood Vaccination: Systematic Review
Source: JMIR Mhealth Uhealth. 2020 May 18;8(5):e17371. doi: 10.2196/17371 (PMC7265109; doi:10.2196/17371)

**Appendix D: Risk of bias assessment**

***Critical appraisal scores using the Critical Appraisal Skills Programme (CASP) Cohort Study appraisal tool***

*
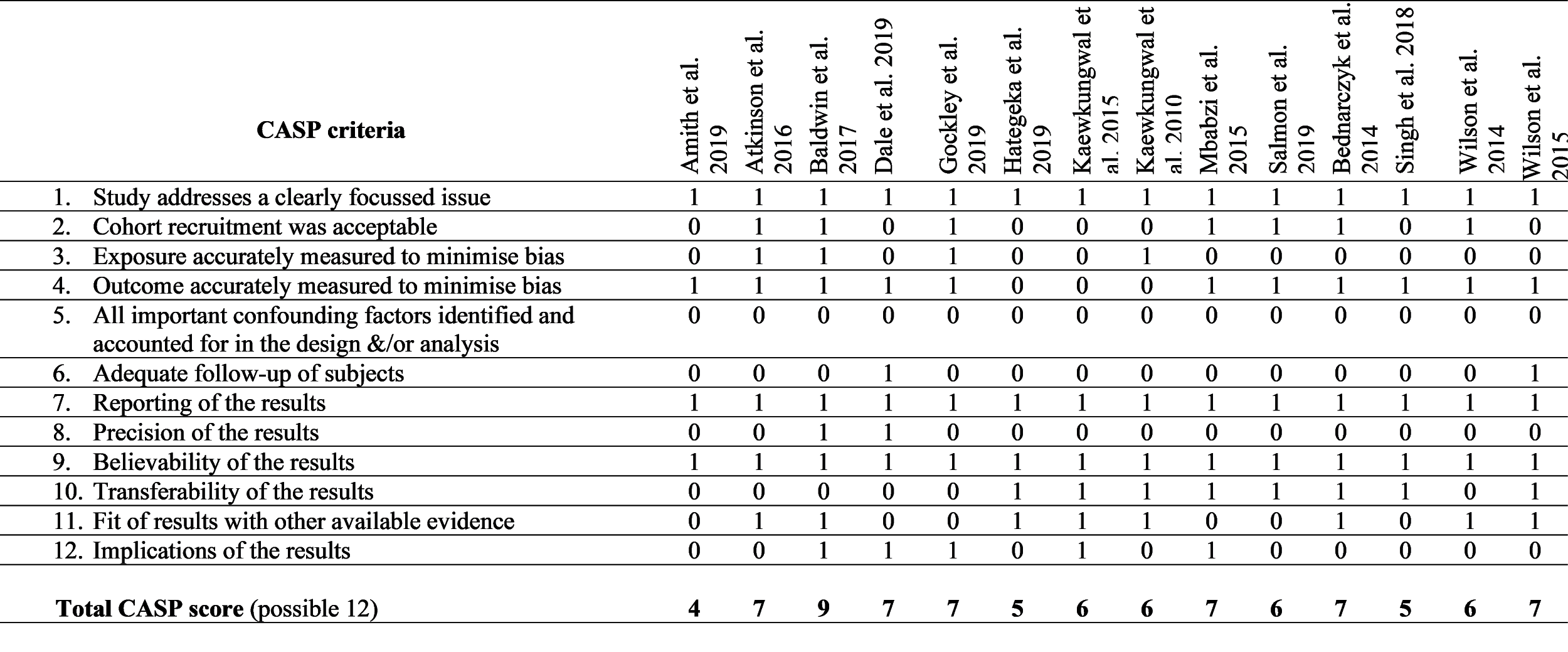
*

***Critical appraisal scores using the Critical Appraisal Skills Programme (CASP) Qualitative Study appraisal tool***

*
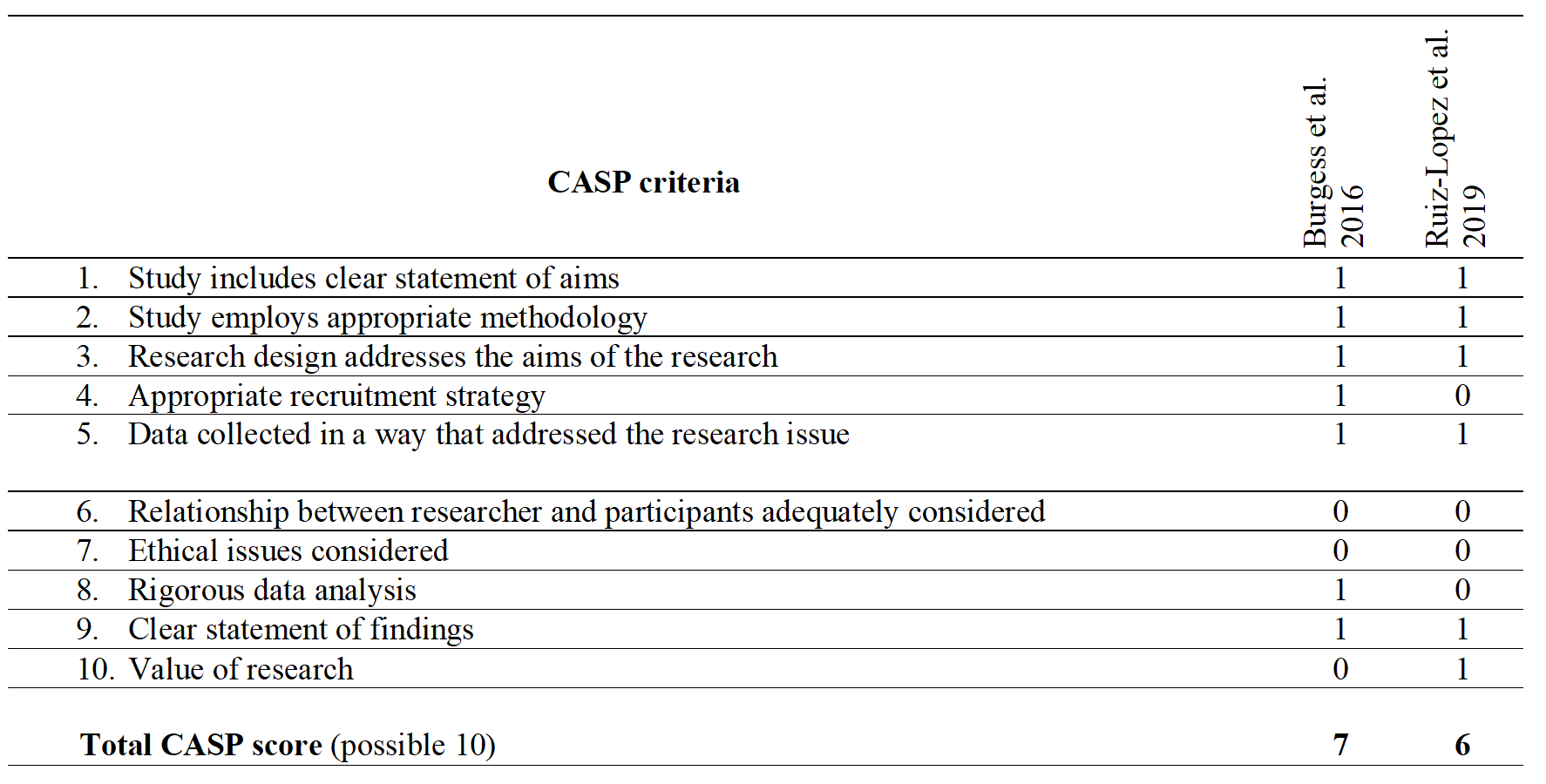
*


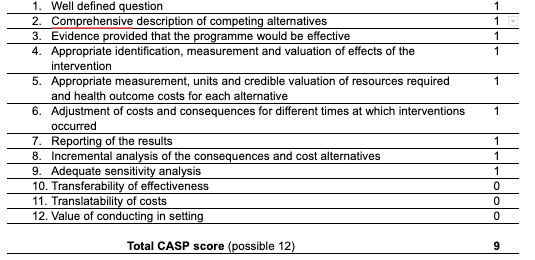
***Critical appraisal scores using the Critical Appraisal Skills Programme (CASP) Economic Study appraisal tool***


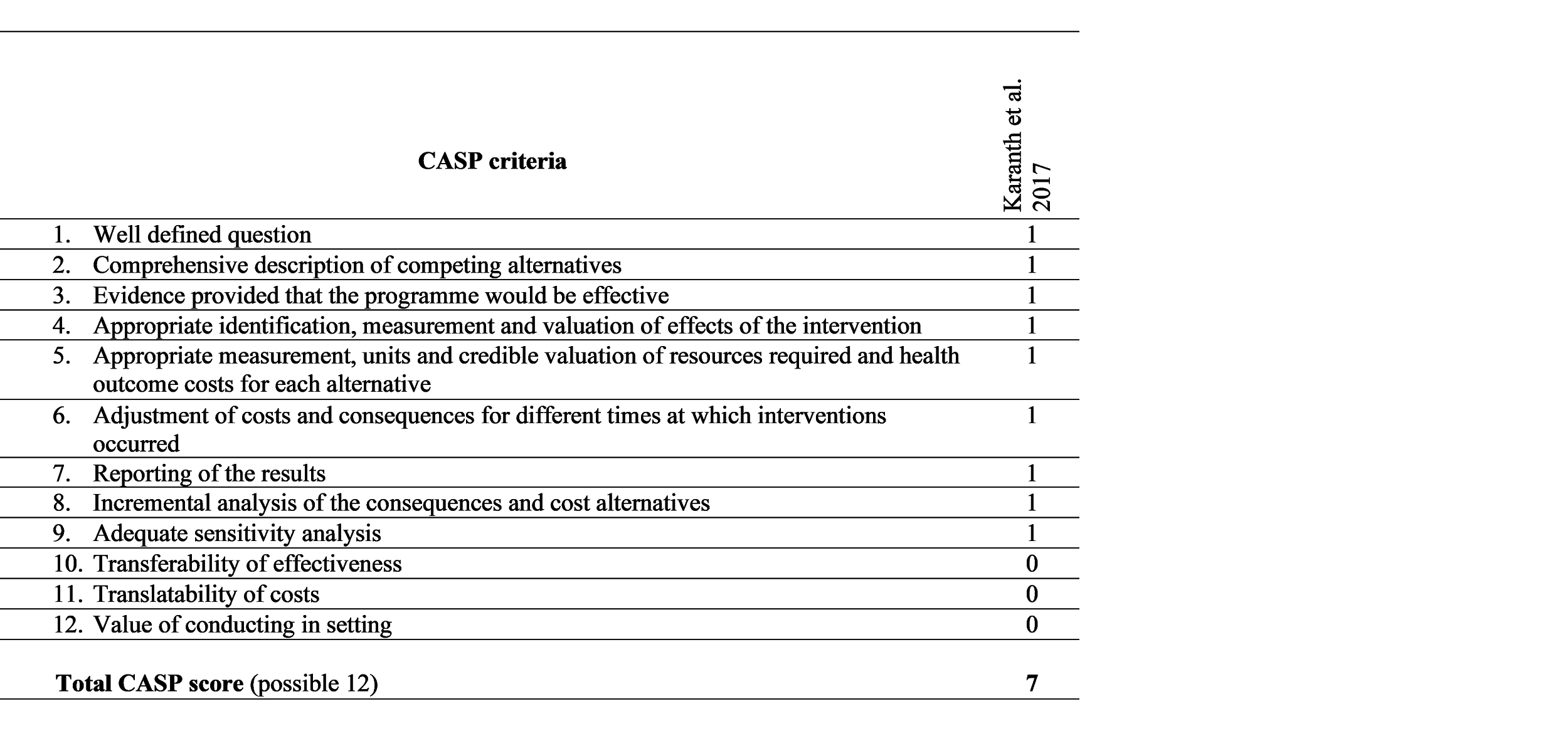


***Critical appraisal scores using the Appraisal tool for Cross-Sectional Studies (AXIS)***


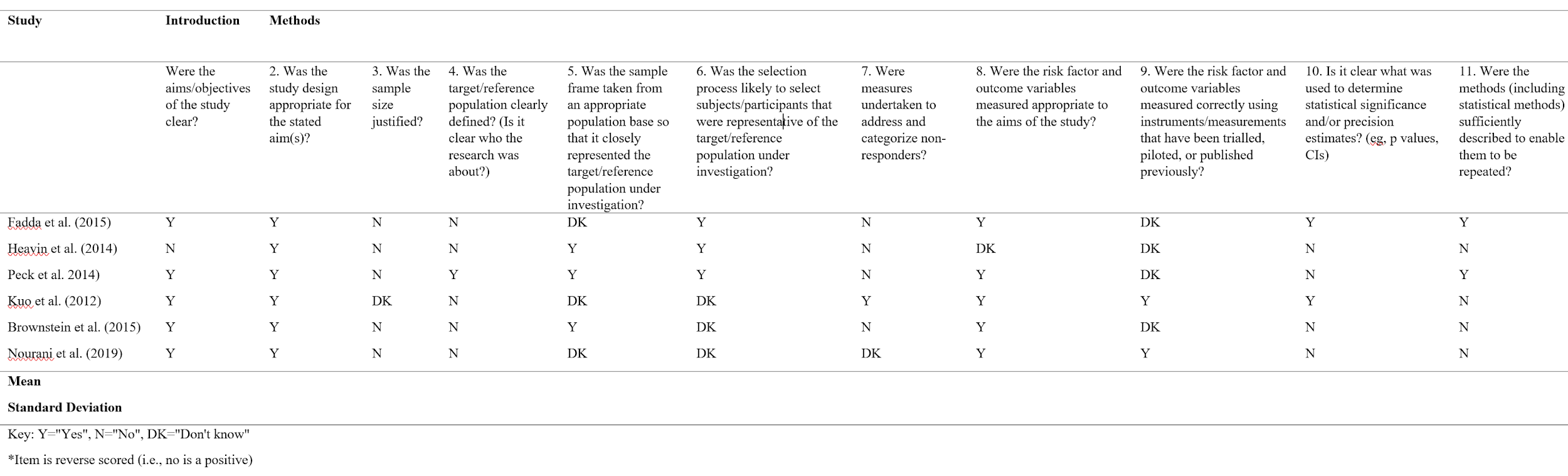

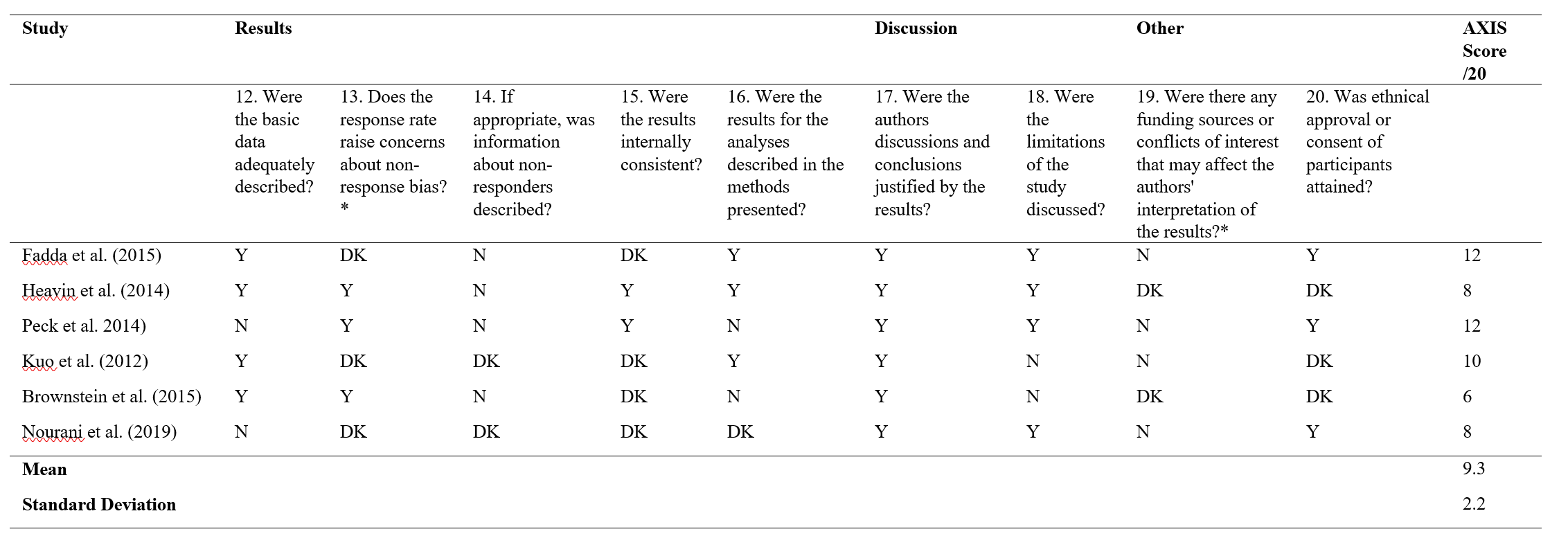


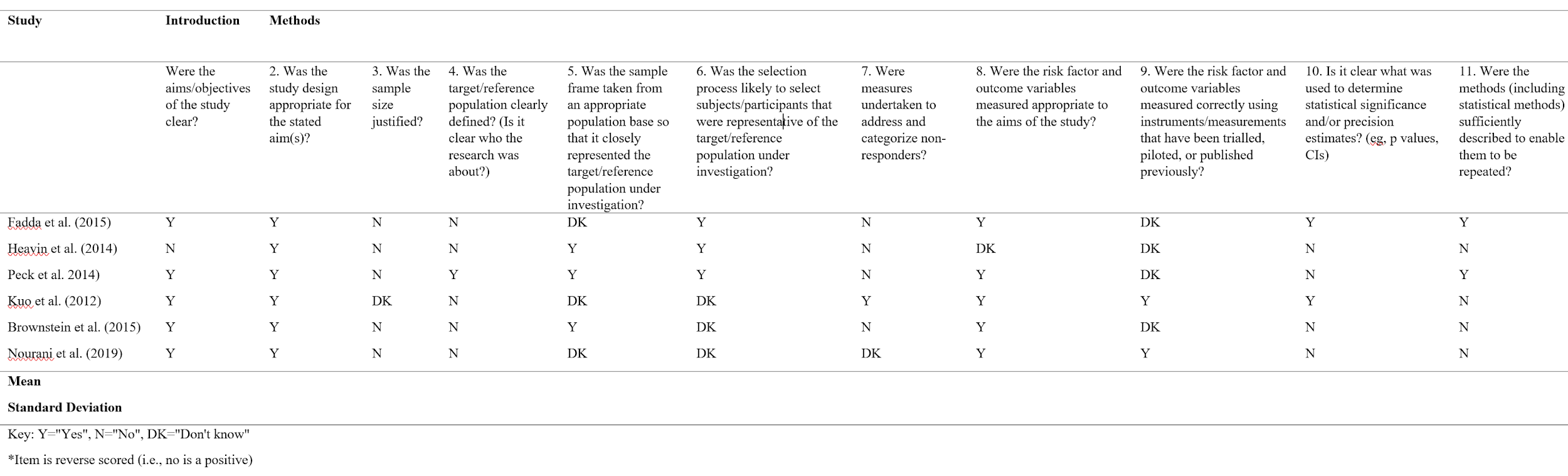


***Robvis traffic light plot describing the ROBINS-I results***


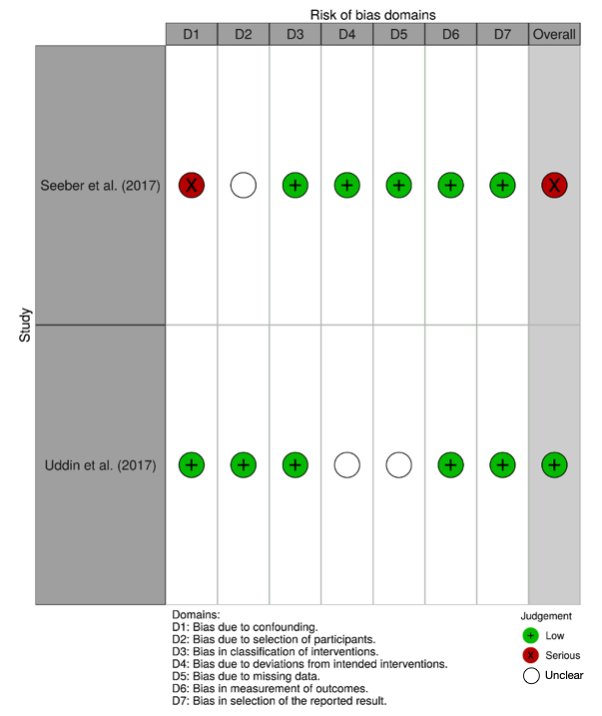


***Revman 5.3 traffic light plot describing Cochrane Collaboration Risk of Bias Tool results***


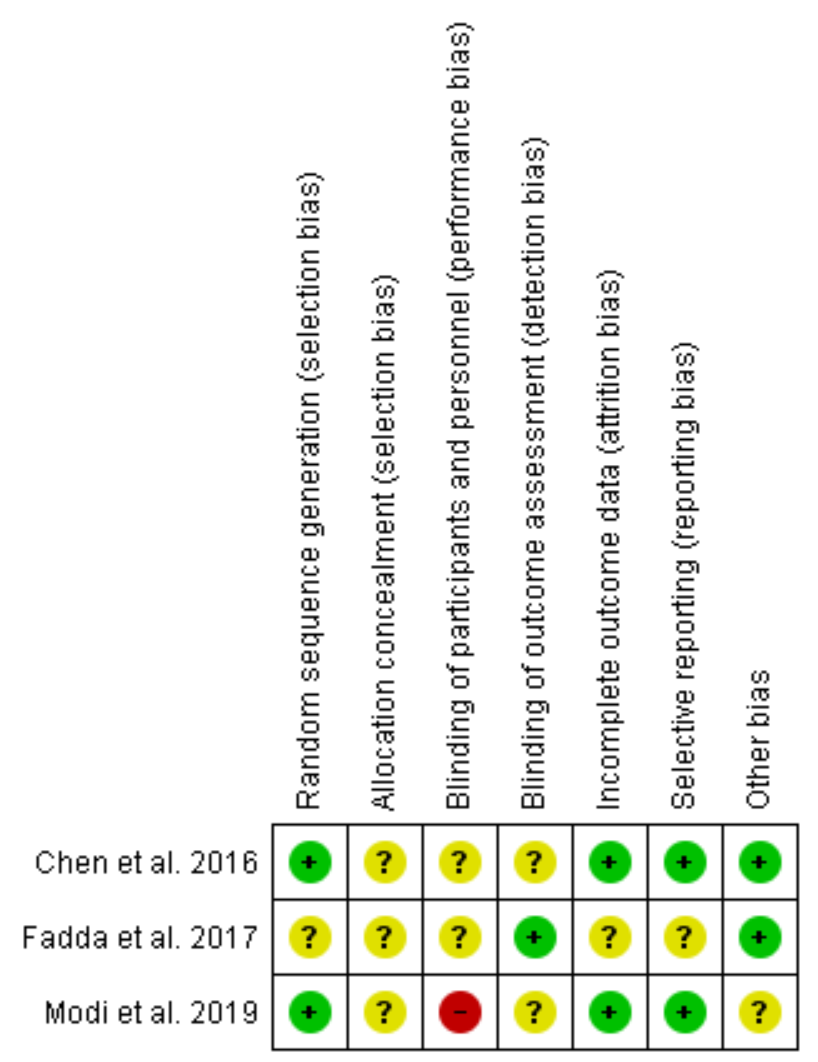

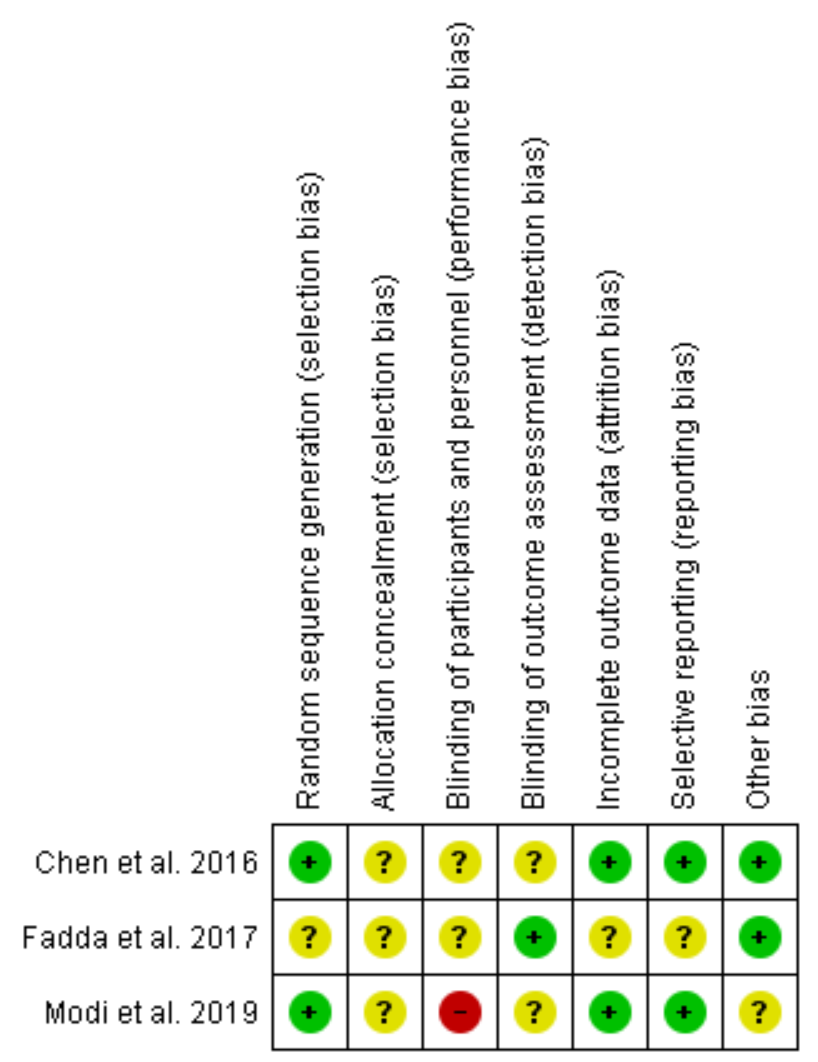


Low risk

Unclear risk

High risk


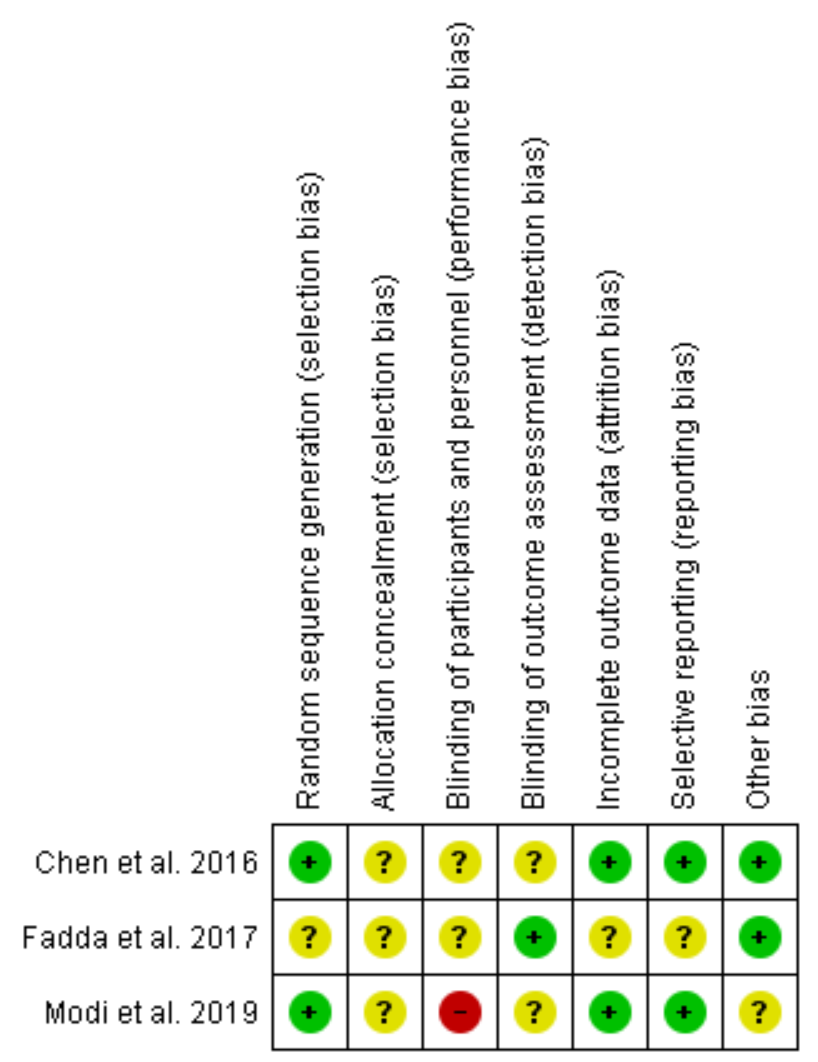

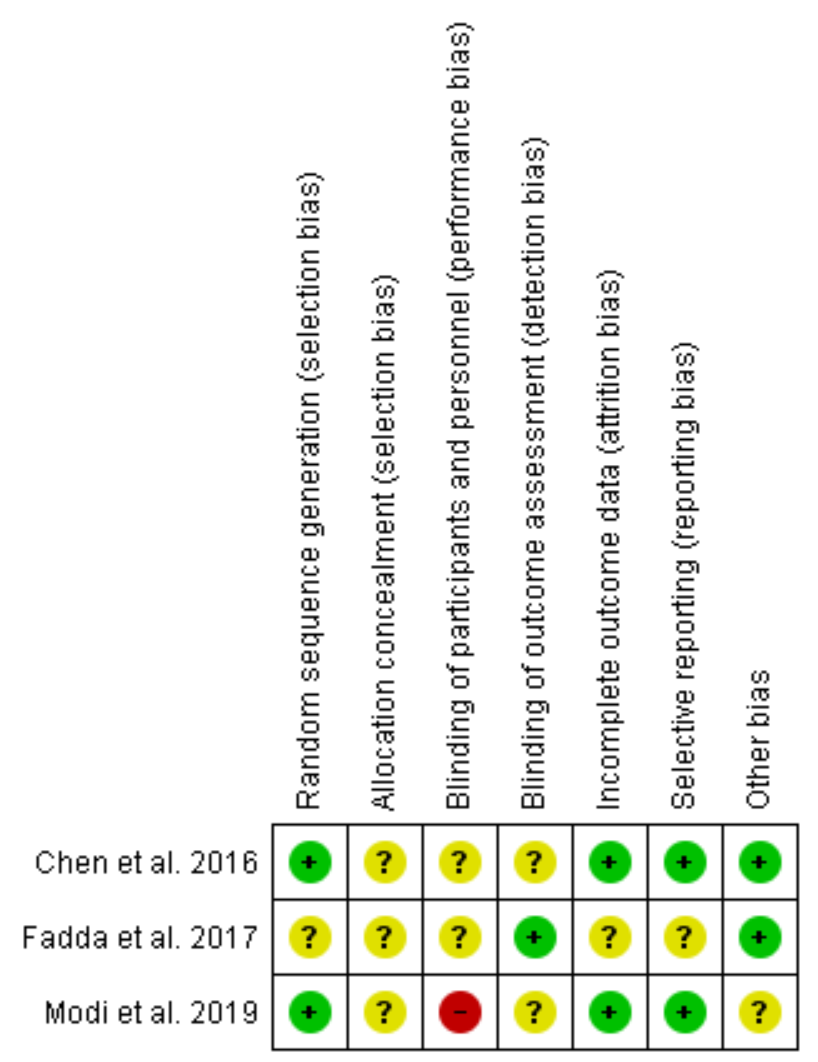

Supplement: Multimedia Appendix 4 [file mhealth_v8i5e17371_app4.docx]
